# Fragile X syndrome: panoramic radiographic evaluation of dental anomalies, dental mineralization stage, and mandibular angle

**DOI:** 10.1590/1678-775720160170

**Published:** 2016

**Authors:** Aida Sabbagh-Haddad, Denise Sabbagh Haddad, Edgard Michel-Crosato, Emiko Saito Arita

**Affiliations:** 1 Associação Paulista de Cirurgiões-Dentistas, Departamento de Odontologia para Pacientes com Necessidades Especiais, São Paulo, SP, Brasil; 2 Universidade de São Paulo, Faculdade de Odontologia, Departamento de Estomatologia, Disciplina de Radiologia, São Paulo, SP, Brasil; 3 Universidade de São Paulo, Faculdade de Odontologia, Departamento de Odontologia Social, São Paulo, SP, Brasil

**Keywords:** Fragile X syndrome, Intellectual disability, Tooth abnormalities, Panoramic radiography, Dentistry

## Abstract

Fragile X syndrome (FXS) is a disorder linked to the chromosome X long arm (Xq27.3), which is identified by a constriction named fragile site. It determines various changes, such as behavioral or emotional problems, learning difficulties, and intellectual disabilities. Craniofacial abnormalities such as elongated and narrow face, prominent forehead, broad nose, large and prominent ear pavilions, strabismus, and myopia are frequent characteristics. Regarding the oral aspects, deep and high-arched palate, mandibular prognathism, and malocclusion are also observed.

**Objective:**

The purpose of this study was to evaluate the dental radiographic characteristics as described in 40 records of patients with panoramic radiography.

**Material and Methods:**

The patients were in the range of 6–17 years old, and were divided into two groups (20 subjects who were compatible with the normality standard and 20 individuals diagnosed with the FXS), which were matched for gender and age. Analysis of the panoramic radiographic examination involved the evaluation of dental mineralization stage, mandibular angle size, and presence of dental anomalies in both deciduous and permanent dentitions.

**Results:**

The results of radiographic evaluation demonstrated that the chronology of tooth eruption of all third and second lower molars is anticipated in individuals with FXS (p<0.05). In this group, supernumerary deciduous teeth (2.83%), giroversion of permanent teeth (2.31%), and partial anodontia (1.82%) were the most frequent dental anomalies. In addition, an increase was observed in the mandibular angle size in the FXS group (p<0.05).

**Conclusion:**

We conclude that knowledge of dental radiographic changes is of great importance for dental surgeons to plan the treatment of these individuals.

## INTRODUCTION

Fragile X (Martin-Bell) Syndrome (FXS) is an inherited genetic disease, which is little known by most professionals in the health area. For this reason, its actual incidence in the population is still unknown although its prevalence is known to be high. Recent studies have shown a pre-mutation prevalence in men (1:430) and women (1:209) in the USA^16^.

The FXS designation is related to a fragile region of the gene, which is located at the distal portion (Xq27.3) of the X-chromosome long arm. The fragile region is a gap, which is not stained by cytological dyes and usually involves both chromatids at a point where the chromosome is susceptible to break. This characteristic can be confirmed by karyotype examination, since the specific investigation technique for fragile sites is used. In men, the diagnosis is made indirectly through the polymerase chain reaction (PCR) method. In women, however, this method is not sufficient to indicate mutation in the gene, and the use of Southern Blot and Hybridization methods is necessary for final diagnosis of the syndrome^1^.

Molecular studies identified this mutation in the Fragile X Mental Retardation type-1 (FMR1) gene, which is located on the X chromosome, and explains the fragile site in the subterminal portion of its long arm. Variable effects can be observed in the phenotypic constitution of individuals with the syndrome due to a gene permutation and expansions observed in the Fragile X Mental Retardation Protein (FMRP) (greater or smaller amount of CGG nucleotides)^6,9^. The alteration in the FMRP is repeated in body cells, affecting various organic structures and functions, mainly those linked to the cognitive ability. Thus, intellectual disability is the most important clinical manifestation, which is caused because the FMRP is absent in the brain of these patients^18,19^. The degree of intellectual disability is extremely variable, even among individuals from the same family. However, severe deficiency seems to be the most frequent manifestation, which occurs in 42.0% of men affected by this mutation^7^.

Given the variable clinical aspects, the consensus in the literature is that the chromosomal or molecular study of individuals with intellectual disability of unknown origin is mandatory to identify the individuals affected by the FXS mutation^1,6,7,9,16,18,19^.

In the FXS, the typical clinical presentation shows a classic triad, formed by macroorchidism (in men), large and prominent ear pavilions, and elongated and narrow face^7^. In these individuals, the face is longer because their mandibles suffer a downward rotation^4^. Their cephalic perimeter is increased and the bizygomatic diameter and internal intercanthal distance are diminished. In addition, their height, wingspan, and length of hands, feet, and digits are also significantly increased^2^.

The behavioral characteristics present in the FXS include poor eye contact, flapping hands, defensive physical contact, and impulsivity, as well as hyperactivity aggressiveness, anxiety, and self-mutilation^5,13^.

The oral and facial clinical examination is a priority in Dentistry, but few studies were found in the databases addressing the oral manifestations of the FXS. However, some authors cite deep and high-arched palate and prominent jaw as the main characteristics of FXS^5,11^. Furthermore, the presence of macroglossia, partial anodontia, stains and enamel hypoplasia, shape anomalies, macrodontia, and unilateral and bilateral crossbite have also been reported^13^.

The purpose of this study was to evaluate the dental structure alterations, mandibular angle measurements, and dental mineralization stage through panoramic radiography, in individuals with fragile X syndrome.

## MATERIAL AND METHODS

This study was approved by the Institutional Review Board (CAAE 46419215.7.0000.0075) of the School of Dentistry, University of São Paulo (FOUSP).

Forty clinical forms were selected. They included panoramic radiographic exams of individuals aged between 6–17 years. These forms are part of the database of the author's private clinic.

The sample was divided into two groups: 20 patients diagnosed with FXS confirmed by molecular analysis (method of double digestion of genomic DNA by the EcoRI and Eagle enzymes, followed by Southern blot and hybridization with the StB12.3 probe), named FXS group and 20 individuals compatible with the normal pattern, which were called control group. Both groups were matched for gender and age, including individuals with deciduous, mixed, and permanent dentition. All of them had anamnesis, oral clinical examination, and panoramic radiographic examination.

**Figure 1:**
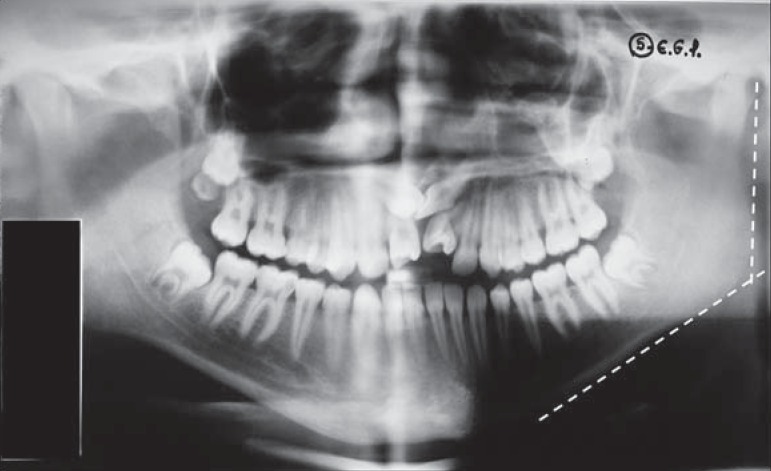
Panoramic radiography with the mandibular angle lines

By panoramic radiographic evaluation (Figure 1), two radiologists analyzed the mandibular angle size, chronology of eruption according to Nolla's criteria^10^, and the dental anomalies related to changes in shape, volume, position such as macrodontia, microdontia, fusion, gemination, concrescence, taurodontism, root fusion and laceration, dens in dente, transposition, giroversion, imperfect amelogenesis, partial anodontia, dental, root supernumerary, dental number of all deciduous and permanent teeth, whether erupted or not. The *kappa and intraclass correlation (ICC) coefficients* were used to test the intra- and inter-rater reliability.

Nolla^10^ calculated the dental mineralization stage dividing the development of each tooth in 10 stages, since the presence of dental crypt until the complete formation of the apex^10^. The author separated tables for men and women, where the average calcification stages were recorded for each tooth in the age range of 6–17 years.

The panoramic radiographs were analyzed using a negatoscope, and the dental mineralization stages were interpreted using the Nolla's 10-stage diagrams of dental development^10^.

Measurements of the mandibular angle were obtained by the intersection of linear measurements tangential to the mandible ramus and inferior border.

All data were compiled into a spreadsheet using the Office Microsoft Excel (Microsoft Corporation, Redmond, WA, USA) program. The SPSS 19^®^ (IBM Corporation; Armonk, NY, USA) program was used to obtain the descriptive statistics, Pearson's correlation coefficients, and Student's *t* test. Differences, associations, and correlations were considered significant when the test descriptive level (*p*) was lower than 0.05.

The relationship between measurements was evaluated by the Pearson's correlation coefficient (*p*), which was evaluated when significant correlations were found. Absolute *p* values suggest weak (|*p*|<0.4), moderate (0.4≤|*p*|<0.7), and strong (|*p*|≥0.7) correlations.

## RESULTS

The panoramic radiographs were analyzed by two observers. Kappa coefficient was used to test intra- and interobserver agreement in all dental anomalies. ICC was used to test intra- and inter-rater reliability in the Nolla stage and mandibular angle measurements. Both coefficients indicate strong correlation (>0.8) between the parameters analyzed.

In most panoramic radiographic exams of our sample, the age group included both deciduous and permanent dentition. Thus, we evaluated separately the frequency of dental anomalies in the deciduous and permanent dentitions in both control and FXS groups (Table 1). The control group did not show dental anomalies in the deciduous dentition (n=138). However, some alterations were present in the permanent dentition (n=608): microdontia (0.16%), root laceration (0.82%), giroversion (0.99%), partial anodontia (0.49%), and supernumerary roots (0.99%). In the deciduous dentition (n=106), the FXS group presented giroversion (0.94%) and supernumerary tooth (2.83%). In the permanent dentition (n=605), we observed fused roots (0.99%), root laceration (1.16%), giroversion (2.31%), partial anodontia (1.82%), and supernumerary roots (0.33%).

**Table 1:** Frequency of dental anomalies (*per* tooth) in the FXS and control groups

Dental Anomalies	Deciduous teeth		Permanent teeth	
	Control group (n=138)	FXS group (n=106)	Control group (n=608)	FXS group (n=605)
	%	%	%	%
Macrodontia	0	0	0	0
Microdontia	0	0	0.16	0
Fusion	0	0	0	0
Gemination	0	0	0	0
Concrescence	0	0	0	0
Taurodontism	0	0	0	0
Fused roots	0	0	0	0.99
Laceration roots	0	0	0.82	1.16
Dens in dente	0	0	0	0
Transposition	0	0	0	0
Giroversion	0	0.94	0.99	2.31
Amelogenesis imperfecta	0	0	0	0
Partial anodontia	0	0	0.49	1.82
Supernumerary tooth	0	2.83	0	0
Supernumerary root	0	0	0.99	0.33

We evaluated all panoramic radiographic exams for eruption chronology by the Nolla's approach^10^, separated the two groups, and observed that individuals with the FXS showed accelerated eruption in the upper (18, p=0.063; 28, p=0.024) and lower (38, p=0.033; 48, p=0.026) third molars, and lower second molars (37, p=0.004; 47, p=0.001).

The bilateral mandibular angle measurements were evaluated and the FXS group showed an increase (8°) statistically significant as compared with the control group (*p*<0.001; Table 2).

**Table 2:** Measurements of mandibular angles in patients of the control and fragile X syndrome (FXS) groups

Groups	n	Right side		Left side	
		M ± SD	Min-Max	M ± SD	Min-Max
Control	20	122.7 ± 5.7	112–132	123.0 ± 6.3	108–136
FXS	20	131.8 ± 5.9	120–144	130.4 ± 7.6	120–142

p<0.001; M: mean values; SD: standard deviations

The Pearson's test for correlation between age and mandibular angle indicated a weak correlation (*p* =0.44) (Table 3).

**Table 3:** Pearson's test for correlation between age and left and right mandibular angles

	Age	Right angle	Left angle
Age	1		
Right angle	−0.123*	1	
Left angle	−0.114	0.792	1

* p =0.449

## DISCUSSION

The fragile X syndrome is a genetic disease with a great variability in clinical presentation. Until the 1990s, this syndrome was diagnosed by clinical signs and chromosomal study (karyotype). However, recent studies have confirmed that the PCR method is not sufficient to indicate mutation in the FMR1 gene in women affected by the disease. Since this discovery, the molecular exam has been included for final diagnosis of the syndrome^1,6,7,9,16,18,19^.

Due to the FMR1 gene permutation and FMRP expansions, variable effects have been observed in the phenotypic constitution of syndromic patients^6,9^.

The radiographic examination is one of the most affordable complementary examinations used in Dentistry for diagnosis, planning, and implementation of treatment, being useful in all dental specialties. Panoramic radiographs are among those examinations, being a part of dental surgeon routine due to the operational simplicity of the equipment, low-dose radiation, low cost, yet allowing examination of a large area of the maxilla and mandible. Furthermore, it is widely used in epidemiological studies in the evaluation of dental injuries and anomalies, whose knowledge is of great value for studies in certain populations.

Analysis of digital panoramic radiographs (1937) of individuals aged 10–34 years showed that dental absence by tooth extraction, partial anodontia, extrusion, migration, transposition, giroversion, and carious and periapical injuries were the most frequent injuries and alterations, with higher prevalence in women. The ones less common in this group were: changes in the condylar head, hypercementosis, mandibular fracture, odontoma, dentigerous cyst, keratocystic odontogenic tumor, cement-bone periapical dysplasia, foreign body, and cleft palate^17^.

Isolated cases of dental radiographic evaluation of FXS have also been published. One study identified the presence of mesiodens and taurodontism in the upper first and second permanent molars^8^. Other author reported the presence of non-erupted supernumerary tooth in the apical region of the upper right central incisor, without change in the eruption chronology^4^.

Individuals with FXS show specific characteristics, such as low caries prevalence, problems of cross and open bite, severe occlusal wear and dental changes including impacted canine, congenital absence of premolar, premolar supernumerary, and a large hypoplastic defect in a tooth alone, as compared with normal individuals^15^.

In our study, supernumerary deciduous teeth (2.83%), giroversion (2.31%), partial anodontia (1.82%), lacerated (1.16%) and fused roots (0.99%), and supernumerary root (0.33%) in the permanent teeth were the most frequent dental anomalies in the FXS group.

As observed in our study, partial anodontia is a disorder in which there is a failure in the dental development of deciduous or, more often, permanent dentition. Partial anodontia is associated with certain disorders such as ectodermal dysplasia, Down's syndrome, and cleft lip and palate^14^. This association also occurs in supernumerary teeth related to the Gardner syndrome and cleidocranial dysplasia, which is caused by an autosomal dominant gene. Although the exact prevalence of isolated hypodontia or supernumerary teeth is unknown, in many cases there is a familial tendency for this defect. It results from mutations in the polygenic system, which is most often transmitted in an autosomal dominant manner, with incomplete penetrance and variable expression. Although FXS is an inherited genetic disease, these radiographic findings cannot be attributed to specific features of the syndrome. Further studies should be done to confirm these findings.

In all studies involving the fragile X syndrome, the relationship between age and mandibular angle was not investigated. Measurement of the mandibular angle, which is the angle between mandibular body and ramus, has been used as a tool to determine the age of individuals. In the range of 3–13 years, age is inversely proportional (in degrees) to the angle^12^. According to our study, individuals with the fragile X syndrome exhibit an 8-degree increase in the mandibular angle when compared with the normal range (p<0.05). However, Pearson's correlation test indicated a weak correlation between age and the measures of mandibular angle in both groups. This age group was chosen based on the mandibular bone growth phase. However, the age range, compared with the sample size, is far too wide (6–17 years) to allow a definitive conclusion on the increase in mandibular angle with age.

Some classic facial characteristics in individuals with FXS, such as long face^4,7^, downward mandibular rotation^7^ and skeletal open bite^14^, do not meet our results regarding the increase in the mandibular angle. Our results suggest that the long face observed in the patients with FXS^2,4,7,15^ could explain the increase in mandibular angle. Further evaluation of cephalometric studies is necessary to confirm the above mentioned hypothesis.

The dental development and its eruption chronology may be influenced by a number of factors such as ethnic group, gender, diet, systemic diseases, infectious processes, climate, and constitutional types. Although dental eruption is influenced by genetic and environmental factors, in most cases it keeps a certain pattern, which can be applied in legal medicine to estimate the chronological age of individuals without an identification document as well as in the dental treatment planning.

Kotilainen and Pirinen^3^ (1999) evaluated the dental development of 28 boys (aged 4.9–17.6 years) with FXS and three girls (aged 5.8, 10.4, and 12.7 years) who were FXS carriers. They used the Demirjian and Goldstein (1976) criteria for tooth development and those of Hagg and Taranger (1985) for tooth eruption, and compared the stature data and bone maturity growth of the individuals. They concluded that the clinical dental eruption of deciduous and permanent teeth in men with FXS was precocious as compared with that observed in controls of the same age. The dental calcification stage was anticipated in men and heterozygous carrier women, and the height and bone maturity growth did not show an anticipated development. Our results were different from those of Kotilainen and Pirinen^3^ (1999) regarding dental calcification, which was anticipated, and dental eruption, which was precocious in all teeth, because the evaluation criteria were different.

## CONCLUSION

Individuals with the fragile X syndrome showed a higher frequency of dental anomalies (supernumerary deciduous teeth (2.83%), giroversion (2.31%), partial anodontia (1.82%), lacerated roots (1.16%), fused roots (0.99%), and supernumerary root (0.33%) in the permanent teeth) when compared with the control group. Additionally, an increase was observed in the mandibular angle and acceleration in the eruption chronology of upper and lower third molars and lower second molars.

Dental surgeons should consider the changes in these findings to obtain a better dental planning and treatment in individuals with the fragile X syndrome.

We suggest that panoramic radiography is included in the usual skeletal radiographic analysis of individuals with FXS to better analyze their mineralization stages, dental anomalies, and mandibular angle measurements and thus better classify their phenotypes.
